# From Assessment to Impact: How Workplace Health Screening Informs Staff Wellbeing in a University Setting

**DOI:** 10.3390/healthcare14070912

**Published:** 2026-04-01

**Authors:** Rebecca Louise Jones, Georgia Clay, Tina El-Bikam, Melissa J. Bargh

**Affiliations:** 1 Institute of Education, Arts and Community, Federation University, Ballarat, VIC 3350, Australia; 2 Physical Activity, Sport and Exercise (PASE) Research Group, School of Psychology, Sport Science and Wellbeing, University of Lincoln, Lincoln LN6 7TS, UK; gclay@lincoln.ac.uk (G.C.); telbikam@lincoln.ac.uk (T.E.-B.); mbargh@lincoln.ac.uk (M.J.B.); 3 Academic Rheumatology, Division of Injury, Recovery and Inflammation Sciences, School of Medicine, University of Nottingham, Nottingham NG7 2UH, UK; 4 Arthritis UK Pain Centre, University of Nottingham, Nottingham NG5 1PB, UK; 5 NIHR Nottingham Biomedical Research Centre, University of Nottingham, Nottingham NG7 2UH, UK

**Keywords:** cardiovascular health, metabolic health, diabetes, health checks, occupational health, community

## Abstract

**Background/Objectives**: With increasing requirements for health assessments, the working environment offers an effective setting to engage individuals at risk of disease. This study aimed to investigate the current health status of university employees through individual workplace health screening and explore the perceived feasibility, relevance, demand and impact. **Methods**: A total of 156 individuals attended a one-off health assessment (September 2024 and July 2025). Sociodemographic characteristics, body composition (height, weight, body mass index, waist-to-hip ratio, waist-to-height ratio), cardiovascular health (heart rate, blood pressure, electrocardiogram) and fasted capillary blood samples (total cholesterol (TC), high-density lipid cholesterol (HDL-C), glucose) were collected. Individuals were then invited to complete an online questionnaire reviewing their experiences, with 71 (67 qualitative) responses collected. **Results**: Females displayed a higher waist-to-hip (*p* < 0.001, Rank biserial correlation (RBC) = 0.65), waist-to-height (*p* = 0.02, RBC = 0.24) ratio, lower systolic blood pressure (*p* = 0.010, Cohen’s *d* = 0.44) and QRISK^®^3 relative risk (*p* < 0.001, RBC = 0.41). Fasted capillary blood samples noted significantly lower glucose (*p* = 0.020, RBC = 0.25) and TC/HDL-C ratio (*p* < 0.001, RBC = 0.48), with significantly higher HDL-C (*p* < 0.001, Cohen’s *d* = 0.93) in females compared to males. A total of three overarching themes were identified from the qualitative responses in the impact assessment, undertaken 2–10 months following screening: ‘*Positive experiences of health screening’,* ‘*The impact of health screening’ and ‘The future of health screening’.* **Conclusions**: This mixed-methods cross-sectional study with post-intervention survey provides an important discussion of the perceived benefits of workplace health screening. Underpinned by both the quantitative and qualitative outcomes, health screening provided a feasible and well-received approach to support the understanding of health in university staff.

## 1. Introduction

Health inequalities are worsening, linked to the social determinants of health including where people are born, life, and work [1]. In England, there is evidence that life expectancy rates are stalling and even declining in certain demographics [2,3]. Key contributing factors include poor diet, physical inactivity, and obesity, with the COVID-19 pandemic further exacerbating these trends [3]. Evaluating an individual’s health and the impact of these modifiable risk factors through preventative screening has been identified as an important approach for increasing awareness of personal risk, supporting early identification of disease, and encouraging timely behaviour change [4,5]. That said, evidence indicates that general health checks have little or no effect on all-cause or disease-specific mortality [6]. It is therefore critical that population health is systematically evaluated and improved. Preventative screening plays an important public health role by identifying modifiable health risks among working-age adults, improving early detection of disease, and extending the reach of preventive services to populations who may not routinely engage with primary care [7,8]. The National Health Service (NHS) has recently been providing health assessments via general practice [9] to target individuals (aged 40–74 years) who might be at greater risk. General health checks are routine health examinations offered to people who appear healthy, focused on spotting risks or diseases early so they can be treated or make changes to their lifestyle [10]. Participating in regular health checks has been found to positively influence factors associated with life expectancy, including higher rates of early diagnosis and preventive interventions [11]. Given that some groups of the public rarely visit their general practitioner (GP) [12], health check screening has now been expanded to additional operating sites such as community pharmacy and occupational health within the workplace [13,14]. In these settings, screening can be conducted and, where necessary, individuals can be referred to general practice to seek guidance on pharmacological treatment and longer-term management [15,16]. Individual experiences in cardiovascular-focused health checks have been described as reassuring (where no risk factors were identified) and a ‘worthwhile shock’ (in the high-risk group) [17].

There are significant factors that can influence engagement in the health check process, including the GP–patient relationship, availability of access, time, and financial resources, impacting the intention and decision to participate in general health checks [10]. Reported barriers include long travel or waiting times, health check financial costs, and mixed interpretation of lifestyle advice (appreciation vs. ‘too preachy’). Providing screening services as a form of occupational health may help overcome such barriers, allowing significant benefits for employees, especially given that the progress in improving health in the workplace has been slower than expected [18].

The workplace is well established as a determinant of health [19] and a positive relationship has been reported between an organisation’s commitment to improving quality of life and a reduction in stress levels and other mental health disorders [20]. It is undeniable that these services do come at a financial cost, with the suggestion that general health checks can only be achieved in a favourable political climate and with the support of various stakeholders who share this common goal [10]. Nevertheless, universities possess significant expertise and human capital, including faculty and students, that can be equipped for community and economic development [21]. Universities therefore represent a unique opportunity to address complex societal challenges by leveraging their interdisciplinary expertise and student engagement [22]. Although health checks are a proven and valuable resource, little is known about their impact within work-based settings, particularly universities. Furthermore, no studies have explored employees’ perceptions of such interventions in these contexts. Therefore, this study adopts an exploratory design aimed at describing the health and wellbeing status of university employees through individual health checks with sex-based comparisons included to contextualise the sample, and to examine the perceived impact and value of these one-off sessions on awareness and engagement with health services.

## 2. Materials and Methods

### 2.1. Study Design

A total of 156 participants volunteered for the health screening session [males: 46 [29%]; females: 110 [71%]] between September 2024 and July 2025, with 71 individuals following up with submission of impact data and with 67 providing qualitative responses. Utilising a mixed-methods approach enabled insight into the participants’ subjective experiences and perceptions of the health screening session and thus, better understanding of its impact. Impact data was collected 2–10 months post-screening, with no repeat physiological measures recorded. The study was conducted in accordance with the Declaration of Helsinki and approved by the Institutional Review Board of the University of Lincoln (protocol code 18528 (30 September 2024) and 19548 (15 November 2024)). Informed consent was obtained from all participants involved in the study. No members of the public were involved in the planning, design, data collection, analysis or interpretation of results for this study.

### 2.2. Experimental Visit

Participants attended one health screening session, where consent was requested for data to be shared with the authors. Participants were free to withhold consent to no detriment to the health screening service provided. Sociodemographic information was collected including date of birth, gender, and postcode of residence. Health screening questionnaires were completed prior to the assessment and checked by the lead investigator (R.L.J) to ensure no medical conditions were present that might affect screening. Participants completed a series of health and wellbeing questions to assess physical activity (International Physical Activity Questionnaire—Short Form; IPAQ-SF), occupational sedentary time (Occupational Sitting and Physical Activity Questionnaire; OSPAQ), anxiety (State Trait Anxiety Inventory; STAI), sleep (Pittsburgh Sleep Quality Index; PSQI) and mood state (The Brunel Mood Scale Questionnaire; BRUMS). Prior to screening, participants were asked to refrain from alcohol and moderate-to-vigorous physical activity for 24 h, with caffeine prohibited on test days. Compliance with these requests was verbally confirmed before the session.

Following a quietly seated 10 min period of rest, resting heart rate (HR), blood pressure (systolic [SBP] and diastolic [DBP]) and 30 s single lead electrocardiogram (ECG) assessments were conducted. Measurements were taken using the Omron Complete monitor (Model HEM-7320T-E3, Omron Healthcare Co., Ltd., Kyoto, Japan) from the participant’s left upper arm at chest height, while the participant was seated with arms on the table, and feet flat on the floor. Recording of bipolar Lead I rhythm strip was achieved by touching the electrodes located on the top face (thumb) and both sides of the monitor (fingers).

Anthropometric measures were then taken including body mass (Seca 770 digital scale, Seca GmbH & Co. KG, Hamburg, Germany) and stature accurate to 0.1 cm (Leicester Height Stadiometer, Seca GmbH & Co. KG, Hamburg, Germany). Stature and body mass values were later employed to calculate body mass index (BMI; body mass [kg]/stature [m]^2^). Body composition was assessed using waist and hip circumference to calculate waist-to-hip ratio (WHR) and waist-to-height ratio (WHtR).

Fasted finger-prick capillary whole blood samples were obtained following an overnight fast (>8 h). A Unistik single-use lancet (Unistik Extra, 21G gauge, 2.0 mm depth, Owen Mumford Ltd., Woodstock, UK) was used, with specimens collected using the capillary tube, then placed on CardioChek’s reagent test strips (35–40 µL sample volume). Samples were immediately analysed using a battery-operated, handheld CardioChek^®^ lipid panel analyzer (Medisave, Dorset, UK) for total cholesterol (TC), high-density lipid cholesterol (HDL-C), TC/HDL-C ratio and glucose according to the manufacturer’s instructions. Calibration and quality control were undertaken prior to use to ensure the reliability and consistency of the assay results. Control solutions from the manufacturer were used for quality control of the device.

Following the session, participants were contacted via email to complete the impact assessment. A total of 71 participants responded to questions via Microsoft Forms involving both quantitative and qualitative components; of these, 67 provided qualitative responses. Quantitatively, participants were asked to evaluate their overall experience, on a Likert scale of 1 to 5, and the extent to which the health screening increased their awareness of their health and changes made as a result. The qualitative questions asked participants to provide further detail to the quantitative responses provided, how the screening has influenced their health behaviour, actions taken to improve their health, wellbeing and lifestyle, and any other suggestions they had to improve the service offered.

### 2.3. Experimental Measurements

**International Physical Activity Questionnaire—Short Form:** The IPAQ-SF is a nine-question form utilised to measure the physical activity levels of participants over the past 7 days. The IPAQ-SF measures frequency, duration, and physical activity intensity level and allows the calculation of metabolic equivalent (MET) weekly working hours (MET-hours/week) [23]. The World Health Organisation (WHO) [24] suggests adults perform at least 150 min of moderate-intensity activity per week, ~600 MET-min/week (four MET × 150 min), for general health benefits.

**Occupational Sitting and Physical Activity Questionnaire:** The OSPAQ allows participants to indicate the proportion of their working time, on a typical workday in the last 7 days, that they spent sitting, standing, walking, and doing heavy labour or physically demanding tasks. Participants reported the number of hours they had worked in the last 7 days and the number of days they were at work. To calculate the minutes per workday participants spent in these activities, self-reported percentage of time spent in each activity was multiplied by the number of hours worked per day at their place of work. Self-reported accuracy is good for sitting (ICC = 0.84) and standing (ICC = 0.64), moderate for walking (ICC = 0.50) and weak for heavy labour (ICC = 0.28) [25].

**State Trait Anxiety Inventory:** The shortened STAI of 20 items was implemented using a Likert scale (1: not at all, to 5: very much so), examining transient anxiety (including feelings of apprehension, nervousness, and physiological parameters of anxiety such as HR and respiration). STAI scores range from 20 to 80 per subscale, with ≤37 suggesting low anxiety, 38–44 moderate, and ≥45 indicating high anxiety.

**Pittsburgh Sleep Quality Index:** Sleep quality over the previous month was assessed for 19 individual items for seven components of sleep quality: (1) sleep duration; (2) sleep disturbance; (3) sleep latency; (4) daytime dysfunction due to sleepiness; (5) sleep efficiency; (6) overall sleep quality; and (7) sleep medication usage [26]. Each component was scored between 0 and 3 and then totalled (total between 0 and 21), with higher scores indicating worse sleep. A global PSQI score ≤ 5 indicates good sleep quality and >5 indicates poor sleep quality [26].

**The Brunel Mood Scale Questionnaire:** The psychological mood tool contains 24 items assessing feelings in the moment. Descriptors were rated on a 5-point Likert scale, ranging from 0 (not at all) to 4 (extremely), including six subscales: anger, confusion, depression, vigour, fatigue, and tension. Each subscale score is totaled from four questions, thus ranging from 0 to 16 per subscale.

**Blood variables:** In accordance with NHS guidelines, healthy levels of blood variables were classified as <5.0 mmol/L for total cholesterol, HDL-C of >1.0 mmol/L for men and >1.2 mmol/L for women; TC/HDL ratio should be as low as possible, ideally <6 mmol/L. Fasting blood glucose was classified as hypoglycemia (<3.9 mmol/L), normal (3.9 mmol/L to 5.6 mmol/L), pre-hyperglycemia (5.6 to 6.9 mmol/L) and hyperglycemia [diabetes] (>7 mmol/L) in-line with the WHO guidelines [27].

**Body composition:** Classification for BMI was those used by the NIH and WHO for White, Hispanic, and Black individuals, including underweight (18.5 kg/m^2^), normal weight (18.5 to 24.9 kg/m^2^), overweight (25 to 29.9 kg/m^2^), or obese (class I: 30 to 34.9 kg/m^2^; class II: 35 to 39.9 kg/m^2^; class III: ≥40 kg/m^2^) [28]. WHR was defined using the following calculation: waist circumference (centimetres)/hip circumference (centimetres). Individuals were classified as normal (men: <1.0; women: <0.85) or high (men: >1.0; women: >0.85) in-line with the WHO guidelines [29]. WHtR was calculated as waist circumference (centimetres)/height (centimetres), classified using the guidelines from the NICE; individuals were classified as healthy (< 0.50), at increased risk (0.50–0.59) or at high risk (≥0.60) [30].

**Cardiovascular health:** A normal resting HR was defined as 60 to 100 bpm, whilst >100 bpm was defined as tachycardia (fast) and <60 bpm as bradycardia (slow) [31]. ECG assessment recordings were analysed within the Omron Complete Monitor device, which can detect common arrhythmias such as atrial fibrillation, bradycardia, and tachycardia. Blood pressure classifications were made in accordance with the British Hypertension Society (BHS) for clinic blood pressure readings, and aligned with the WHO and the International Society of Hypertension and the European Society of Hypertension [32]: ‘Optimal’—systolic < 120, diastolic < 80 mmHg; ‘Normal’—systolic < 130, diastolic < 85 mmHg; ‘High Normal’—systolic 130–139, diastolic 85–89 mmHg; ‘Grade 1 Hypertension (Mild)’—systolic 140–159, diastolic 90–99 mmHg; ‘Grade 2 Hypertension (Moderate)’—systolic 160–179, diastolic 100–109 mmHg; and ‘Grade 3 Hypertension (Severe)’—systolic ≥ 180, diastolic ≥ 110 mmHg. If SBP and DBP readings fell into different categories, blood pressure was classified according to the higher category. Mean arterial blood pressure (MAP) was determined using the following calculation [33]; *diastolic blood pressure + {[0.33 × (systolic blood pressure − diastolic blood pressure)]}*. MAP was classified as ‘Low’ (≤60), ‘Borderline/Suboptimal’ (61–69), ‘Normal’ (70–100), ‘Elevated’ (101–109) and ‘High–Hypertensive’ (≥110).

The web-based QRISK^®^3 model calculator, developed and validated by Hippisley-Cox et al. [34], was implemented to determine ten-year cardiovascular disease and heart age in adults aged 25 to 84 years. Risk factors included are as follows: age, gender, ethnicity, smoking status, diabetes status, angina or heart attack in a first-degree relative under 60 years old, chronic kidney disease (stage 3, 4 or 5), atrial fibrillation, blood pressure treatment, migraine, rheumatoid arthritis, systematic lupus erythematosus, severe mental illness, atypical antipsychotic medication, steroid use, erectile dysfunction, TC/HDL-C ratio, SBP, height, and weight. Outcomes included ‘Your ten-year QRISK^®^3 score’, ‘the score of a healthy person with the same age, gender, and ethnicity’, ‘relative risk’, and ‘your QRISK^®^3 healthy heart age’.

### 2.4. Data and Statistical Analysis

The sociodemographic characteristics of the participants, with mean and standard deviation of blood variables (TC, HDL-C, TC/HDL-C ratio and glucose), body composition (BMI, WHR, WHtR) and cardiovascular health (HR, blood pressure), were described using descriptive analyses. Independent sample *t*-tests were conducted in Jamovi for Windows (Version 2.4.4, Sydney, Australia). Independent sample *t*-tests were used for the normally distributed data to analyse differences between female and male individuals; a Bonferroni adjustment was implemented to reduce the likelihood of Type I error. Where Levene’s test or Shapiro–Wilk tests suggested a violation in the assumption of equal variance or normality, non-parametric Mann–Whitney U tests were used to analyse differences between the two groups. Cohen’s *d* levels were used to grade the effect size level (low: Cohen’s *d* = 0.2–0.5; moderate: Cohen’s *d* = 0.5–0.8; and high: Cohen’s *d* ≥ 0.8 [35]). All data are presented as mean ± 1 standard deviation (95% confidence interval [CI]) unless stated otherwise. Statistical significance was set at *p* ≤ 0.05.

The qualitative responses from the impact assessment were analysed using thematic analysis [36] by the second author [G.C.]. Qualitative data was collected 2–10 months following post-screening, with no repeat physiological measures recorded. As outlined by Braun and Clarke [36], thematic analysis involved familiarisation with the data upon receipt of the qualitative responses. Semantic coding was undertaken across all responses. Blind, independent coding was also undertaken by the first [R.L.J.] and fourth author [M.J.B.], after which high agreement was reached in how each author had interpreted the meaning of the data represented by the codes. Upon confirming agreement, themes were developed including both subthemes and overarching themes, ensuring that each theme was sufficiently grounded in the data to support the construction of the analytic narrative. The analysis was considered complete when no new codes were developed, and the data was fully represented by previous coding.

## 3. Results

### 3.1. Participant Characteristics

A total of 156 participants were included in the current physiological analysis, with characteristics reported in Table 1. Male and female participants were of similar age, yet males were significantly taller and heavier than females (*p* < 0.001; Table 1). The three most prevalent health conditions reported in family history status was reporting of angina or heart attack in a first-degree relative under 60 years old (22%, nine males, 25 females), reporting of migraines in 34 individuals (22%), and reporting of atypical antipsychotic medication use in 12 individuals (8%; see Table 1). There were 18 different ethnic groups included: African British (n = 1, male = 1), Asian (n = 3, male = 1), British Asian Mixed (n = 1, male = 0), British Indian (n = 1, male = 1), Chinese (n = 2, male = 0), Indian (n = 2, male = 1), Irish (n = 1, male = 0), Mixed White/Black Caribbean (n = 1, male = 0), North African (n = 1, male = 1), South Asian (n = 1, male = 0), Sri Lankan (n = 1, male = 0), Turkish Cypriot (n = 1, male = 0), Vietnamese–Asia (n = 1, male = 0), White British (n = 123, male = 36), White Canadian (n = 1, male = 0), White European (n = 10, male = 4), White Irish (n = 1, male = 0) and White Other (n = 4, male = 1). Data has been reported as overall and for each sex to enhance clarity of the data interpretation.

There were four individuals (three female) classified as ‘underweight’ according to BMI outcomes, 71 as ‘healthy’ (52 female), 49 as ‘overweight’ (34 female), and 32 as ‘obese’ (21 female). BMI outcomes are reported in Table 1, with no significant differences observed between males and females (*p* = 0.13, Rank biserial correlation (RBC) = 0.15). There was a significant difference for WHR between males and females (*p* < 0.001). There were 18 (out of 46) males classified as ‘elevated risk’. Of the 110 females, 18 were classified as having ‘elevated risk’. For WHtR, there were 70 individuals (62 female) classified as ‘healthy’, 61 individuals (41 female) at ‘increased risk’ and 15 individuals (seven female) at ‘high risk’. Females displayed significantly greater WHtR compared to males (*p* = 0.02, RBC = 0.24). WHR and WHtR are reported in Table 1.

Average resting HR was 67 bpm, with 94 of the 156 individuals’ HR classified as ‘great’ (68 female), 18 as ‘good’ (13 female), 29 as ‘above average’ (20 female) and 15 as ‘average’ (nine female; Table 1). There was no significant difference between males and females (*p* = 0.330, Cohen’s *d* = 0.17). SBP, DBP and MAP are reported in Table 1, of which only SBP was significantly different between females and males (*p* = 0.010, Cohen’s *d* = 0.44). Of the 156 individuals, 46 reported ‘optimal’ blood pressure (37 female), with 54 recorded as ‘normal’ (37 female) and 25 as ‘high normal’ (16 female). There was hypertension reported in 31 individuals: 23 at ‘Grade 1—Mild’ (17 female), seven at ‘Grade 2—Moderate’ (two female) and one at ‘Grade 3—Severe’ (one female). Most individuals reported ‘Normal’ MAP (n = 118; 88 female), with an average MAP of 96 ± 10 mmHg. Twenty-three individuals were classified as having ‘Elevated’ MAP (13 female) and 15 were classified as having ‘High–Hypertensive’ MAP (nine female). Normal ECG classifications were reported in 143 individuals (101 female), a further five displayed signs of bradycardia (two female), six were requested to return for a 2nd visit (three female), and two did not complete this assessment (both female).

### 3.2. Health-Related Outcomes

Physical activity levels on average were classified as ‘moderate’ for both males and females, with no significant difference between sexes (Table 2). Within the 156 participants, 24 individuals (17 female) were classified as engaging in ‘low’, 99 (71 female) in ‘moderate’ and 32 (21 female) in ‘high’ physical activity levels. OSPAQ data reported that participants worked an average of 37 h over five days. Females were reported to work significantly fewer hours than males (36.5 vs. 38 h; *p* = 0.035, RBC = 0.21). The percentage and minutes of the workday spent sitting, standing, walking and engaging in heavy labour are reported in Table 2. According to the global PSQI score, 138 (88%) out of the 156 individuals were classified as poor sleepers, of which 100 of these were female. On average, the global PSQI score was 8 ± 2, above the cutoff for ‘good sleepers’ (≤5). Scores ranged from 4 to 16. All BRUMS questionnaire data are displayed in Table 2, including subscales for anger, tension, depression, vigour, fatigue, confusion, happiness and calmness. Of the 156 individuals completing the STAI questionnaire, 144 reported ‘no or low anxiety’ levels, with six recording ‘moderate’ levels (all female) and a further six reporting ‘high’ levels (all female) of anxiety (see Table 2).

### 3.3. Capillary Blood Outcomes

**Figure 1:**
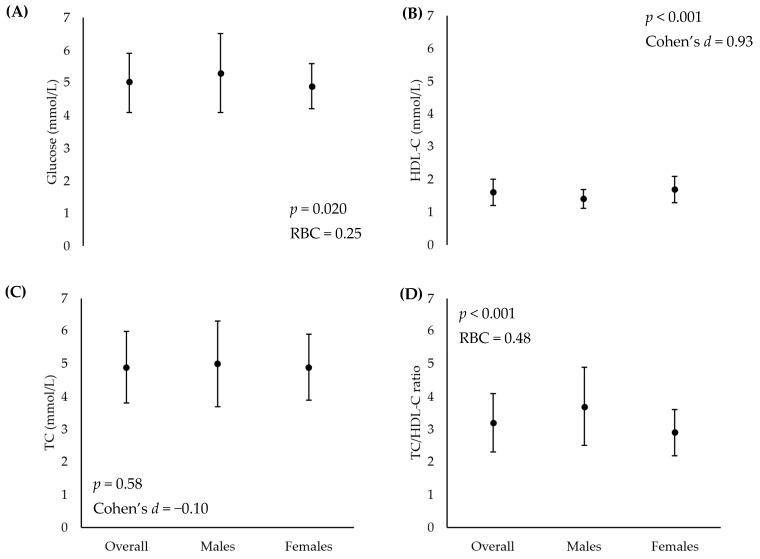
Fasted capillary blood sample analysis; (**A**) glucose, (**B**) high-density lipoprotein cholesterol, (**C**) total cholesterol and (**D**) total cholesterol/high-density lipoprotein cholesterol. TC: Total cholesterol. HDL-C: High-density lipoprotein cholesterol. RBC: Rank biserial correlation. Data presented as mean and 1 standard deviation, with statistical significance set at *p* ≤ 0.05.

Fasted blood glucose concentrations were reported as normal in 112 individuals (78%, 87 females), pre-diabetic for 36 individuals (56%, 20 females), and diabetic for three individuals (0%, n = 0 females). Five individuals did not undertake this test. There was a significant difference between male and female glucose concentrations (*p* = 0.020, RBC = 0.25) (Figure 1). Of the 152 individuals where TC was reported, 86 (57%) displayed ‘great’ classification; of these, 69% (59) were female. Additionally, 49 were classified as ‘above average’ (76%, 37 female) and 17 as ‘high’ (65%; 11 female). There was no significant difference between male and female TC values (*p* = 0.58, Cohen’s *d* = −0.10) (Figure 1). HDL-C was reported as ‘great’ in 119 individuals (78%), including 89 females accounting for 75% of the sample. Classification of ‘good’ was reported in 21 individuals (11 females) and ‘low’ in 12 individuals, of which nine were female. Females displayed significantly higher HDL-C concentrations compared to males (*p* < 0.001; Cohen’s *d* = 0.93) (Figure 1). The TC/HDL-C ratio was classified as ‘great’ for 114 individuals, (75%) of which 80% (91) were female; ‘above average’ classifications were reported in 35 individuals (23%), including 16 females (70%); and classifications of ‘high’ were evident in three male individuals. TC/HDL ratios were significantly lower in females compared to males (*p* < 0.001, RBC = 0.48) (Figure 1).

### 3.4. Qualitative Examination of Impact Data

Of the 156 individuals who completed physiological screening, 71 responded to the impact questionnaire with 67 providing qualitative feedback. The average age of these individuals was 41 ± 11 years; 50 were female (75%) and 17 were male (25%). There were 11 different ethnic groups included: African British (n= 1, male = 1), Asian (n = 2, male = 1), British Indian (n = 1, male = 1), Chinese (n = 1, male = 0), Indian (n = 1, male = 0), Irish (n = 1, male = 0), South Asian (n = 1, male = 0), White British (n = 49, male = 11), White European (n = 6, male = 3), White Irish (n = 1, male = 0) and White Other (n = 3, male = 1).

A total of three overarching themes were identified from the qualitative responses in the impact assessment: ‘*Positive experiences of health screening’,* ‘*The impact of health screening’ and ‘The future of health screening’*. The themes demonstrate the participants’ experiences of how health screening influenced their understanding of their own health, descriptions of lifestyle changes made since the screening, and their perceptions about how such an intervention may have the potential to support current screening efforts of healthcare systems like those offered by the NHS. Each overarching theme is explored in turn, and subthemes are italicised to support identification. Code counts for each subtheme and overarching theme are provided in Supplementary Table S1.

#### 3.4.1. Theme 1: Positive Experiences of Health Screening

Overall, the majority of participants reported having had a highly positive experience of completing the University health screening and felt that overall, the *researchers’ skills were of great benefit to their overall experience.* Participants reported an excellent service, and especially how friendly the researchers were which helped them feel at ease, as Participant 39 (female, aged 31 years) stated “The tests were thorough and the people completing them were nice and friendly and made you feel comfortable”. Approximately a third of participants felt that the level of knowledge and expertise exhibited by the researchers was beneficial, especially for providing interesting insight and information surrounding the screening procedures and were overall very professional. The emphasis upon the overall experience helps to demonstrate the importance of having individuals delivering such screening interventions who are personable and can deliver the relevant information in a way that is easy to understand:

The staff who carried out the checks relayed their knowledge of specifics of the checks in an informative way and the insight was of interest to me. I commend their expertise (Participant 54, female, aged 39 years).

A few participants also commented on the *accessibility of the health screening*, and that having it provided within the workplace made it convenient to access; one participant felt the screening was less intimidating than going to a GP. Overall, the participants felt that the screening was of *benefit as a workplace initiative*, especially as some perceived that if utilised longer term this kind of health screening could play a role in reducing absenteeism and/or presenteeism in the workplace as people may better understand their health:

Screening for a variety of health issues and focusing on preventative measures for optimum health makes so much sense in the context of a workplace. Smaller costs now to reap benefits longer term—less staff off sick and a more healthy and robust workforce (Participant 38, female, aged 47 years).

Just over a fifth of participants emphasised that they felt workplace health needs to be a priority for organisations and that the screening was helpful for them in taking care of their health and welfare. Some also felt the addition of the screening session made them feel valued as an employee, as Participant 33 (male, aged 56 years) recognised “supporting our colleagues to be healthy is a good example of the kind of culture we should be aiming for”. Participants therefore overall conveyed positive perceptions towards the screening in their workplace due to ease of access and the utility of information gained.

#### 3.4.2. Theme 2: The Impact of Health Screening

For most of the participants, they described *the overall positive impact of the screening* on their own health and wellbeing at the time of completing the impact questionnaire. These participants highlighted how the checks provided “a really good insight into my health” (Participant 56, female, aged 50 years) and highlighted that gaining a better understanding of their physical health was useful for them whilst for a few, also potentially helped to detect health concerns. A small number of participants felt the information provided was potentially lifesaving, due to the potential to identify underlying markers of ill health, as Participant 9 (female, aged 38 years) stated “These health checks can potentially save lives and help individuals spot health issues early.” Just under half of participants reported that the screening helped increase their health awareness about their own health, and supported them in being able to better engage with and manage their own health:

The intervention exposed that I had high blood pressures and cholesterol. This is now being manged through medication, and my [levels] are now safe and a fell much better (Participant 6, male, aged 42 years).

Whilst a few participants disclosed that they already had a good understanding of their health prior to their screening appointment, others noted changes made because of the screening and thus highlighted the *personal impact of the screening on employee health*. Around half of participants described either maintaining or implementing new healthy habits and how such changes resulted in perceived improvements to their overall wellbeing. For example, participants reported getting more active after the screening, including cycling to work, strength training, going to the gym and increasing their steps. Some reported implementing better eating habits which resulted in weight loss and reduction in blood pressure readings. Importantly, participants perceived that the screening session encouraged them to monitor their own health more, and helped to motivate them to prioritise their wellbeing and potentially re-focus their personal health goals where needed, as Participant 61 (female, aged 26 years) described

I feel this check was a way of seeing data of these changes and motivates me to keep making the better choices when it comes to diet. I’d assume that if these results had been concerning, I would feel the same motivation to make changes and would feel more aware of my health.

Another insightful subtheme emphasised how participants felt that if implemented longer term, such screening may *help support current health service efforts.* Participants noted that if implemented longer term, a workplace screening service may help address a gap in the current healthcare system in the UK, and may also support participants to prevent ill health before it becomes a problem. Such screening is currently prioritised for anyone above the age of 40 years in the UK, yet those below this age like Participant 57 may have limited access to such services or the opportunity to discuss results face-to-face:

At the age I am, we don’t get offered anything like this on the NHS and I think this would identify where issues are starting to arise before they become more of a health concern. I have recently had my usual check up with my GP scrapped and being asked to input my weight, height and blood pressure data into the system which requires me to attend a pharmacy (Participant 57, female, aged 31 years).

Whilst one participant noted that the screening should not replace GP visits, some felt it could help to support what is currently available across health services especially in relation to the current time and resource pressures facing the NHS, as Participant 44 (female, aged 63 years) describes; “given NHS services are under pressure. it’s a win-win situation for the university and staff.” Participant 40 emphasised how GP appointments are only for when people are feeling ill or have symptoms, whereas the screening could help identify issues before individuals get to this stage:

It’s a good way of picking up potential issues early, less intimidating than going to see the Health Centre doctors. In general, we only do that when already feeling unwell (Participant 40, female, aged 52 years).

#### 3.4.3. Theme 3: The Future of the Health Screening

Overall, participants supported the *continuation of the health screening service* within their workplace, with many reporting that they would recommend the screening to be taken up. A fifth of the participants felt specifically that the screening service is incredibly valuable and should be offered permanently within the university. For example, participants said they would attend the screening again if offered, with some stating they would also pay for the service if it continued in the future and required resourcing. The demand for the service appeared to be high overall, and from participants’ experience of accessing the screening service:

“I understand that it may be very difficult to offer these to all staff, however I definitely believe that the amount available should be increased. Every time these have been run through [university human resource department] there has been massive demand for them” (Participant 52, male, aged 36 years).

In relation to how the health screening could be developed in the future, a fifth of participants feel they would benefit from *opportunities for follow-ups* to their first appointment. Whilst a second appointment was offered to some participants who displayed concerning or irregular results (for example, reassessment of ECG), the qualitative responses indicate a desire for a re-screening opportunity to be offered more broadly to maintain engagement and understanding of their health over time. These participants reported that a follow-up appointment would be beneficial, especially for being able to track any changes and progress resulting from some of the lifestyle amendments they have made as outlined above. For a few individuals, they also thought it would be useful to be able to discuss their results further with those conducting the screening in greater detail:

It did make me realise that my efforts to take care of my health seemed to be working well and feel like another check-in soon would be useful to see if changes that I have been making recently to further take care of my health have made changes since last time (Participant 1, female, aged 28 years).

Finally, some observed *issues and challenges* that should be addressed prior to further development or implementation of this or similar health screening service(s) were identified by participants from their experience of the health screening. The challenges were often reported to be minor, such as technical issues experienced on the day, the appointment feeling rushed and the need for the organisers to reconsider how environmental factors (i.e., walking to the appointment or short-term illness) may influence the results on the day. Three participants also noted receiving a false alarm on some of their results which were later found to be normal in follow-ups with a doctor. These comments emphasise the importance of making participants aware of the limitations presented with one-off screening assessments such as this, as well as the importance of follow-up testing.

#### 3.4.4. Qualitative Impact Data Conclusion

Qualitative analysis of the impact data highlights the relevance of future workplace and/or university health screening, including further development of the present intervention, being conducted by individuals who are approachable and hold sufficient knowledge and expertise regarding the assessments and results, and who are approachable. Having access to such expertise and screening within the workplace setting was considered highly beneficial and participants valued the proximity of the opportunity to their daily environment. Participants particularly felt they benefitted from gaining an increased awareness of their overall health status and detection of early health concerns, thus identifying those longer-term approaches, such as screening interventions, may help to reduce some of the pressures on healthcare services that occur from late diagnosis of chronic conditions. Such insight led to many participants making some changes to their lifestyle, demonstrating the early impact of the screening as observed from the participants. Lastly, future health screening in workplace settings could include follow-up appointments to support motivation and longer-term changes to health-related behaviour.

## 4. Discussion

The current cross-sectional investigation with post-intervention survey is the first to the author’s knowledge to utilise a mixed methodological approach to examine the role of free health screening in supporting employee wellbeing from a university population. Of the 156 individuals, only 71 displayed a ‘healthy’ BMI, 51 reported ‘normal’ blood pressure classifications, and although 143 individuals displayed normal ECG classifications, five displayed signs of bradycardia (two females) and six were requested to return for ^a^ second visit (three females). An important consideration of the current data is the differences in health-based outcomes between males and females of a similar age and physical activity level, with outcomes relating to body composition, cardiovascular health and QRISK^®^3 significantly affected. Qualitative examination of the perceived benefits of free workplace health screening in 67 individuals identified three overarching themes: ‘*Positive experiences of health screening’,* ‘*The impact of health screening’, and ‘The future of health screening’.* This combination of methodologies allowed for greater understanding of the health and wellbeing of the individuals who engaged in this one-off screening session, including their perceived outcomes on their own health, any self-reported lifestyle changes, and their suggestions on how such approaches could be adopted to better support broader healthcare systems. Given the feasible and well-received approach of the current one-off health screening session, such processes could be explored further to help observe and provide an avenue to alter the health of this population, especially given the overall global burden of non-communicable diseases and the current context of preventative health agendas [37].

Targeted screening, specifically for cancer, has been shown to prevent thousands of deaths, with significant long-term benefits [38]. Yet, general health checks, like the current approach, have displayed a mixed outcome; there is currently no clear consensus on mortality and morbidity benefits. A systematic review of 17 randomised trials (≈251,891 participants) noted that there was no evidence of reduced morbidity, hospital admissions, disability, anxiety, or sick leave [39]. Yet, Thonon et al. [40] focused on workplace preventive interventions (including screening) across Europe, finding that ~57% of interventions showed positive return on investment. The current qualitative data suggests that in the current population, both the employee and organisational benefits may be worth the input. Furthermore, the perceptions of free health screening services as an approach to enhance employee wellbeing, provide a free service from an employer and support individuals were noted in the current study. Offering workplace health screening as a strategic employee benefit could be perceived positively, with previous longitudinal research highlighting that workplace health programmes can lead to improved co-worker relationships, enhanced long-term wellbeing and job satisfaction [41]. The current exploratory, cross-sectional investigation with post-intervention survey contributes to the acceptability, feasibility and perceived value of one-off health screening services in this population.

Health screening sessions are now being widely promoted by health services; for example, the NHS Health Check programme (a cardiovascular screening initiative launched in 2009) has been shown to significantly improve detection of hypertension, diabetes, cholesterol, and chronic kidney disease, with uptake higher among women, older adults, and those in higher socio-economic groups [42]. Yet there are some key factors that negatively impact these health checks, including healthcare professionals lacking time and resources and patients facing financial and social barriers, overall reflecting the systemic issues affecting delivery efficiency and access [43]. This is supported by the current qualitative impact data which highlighted the *Accessibility of the health screening*, specifically the convenience of screening being undertaken at the workplace and reducing feelings of intimidation in having to attend more formal screening within GP services. With individuals missing or delaying important health screening sessions, and thus potential diagnoses, there are key structural and equity-related implementations linked to health disparities, poorer outcomes, and increased burden on affected individuals [44]. Cheraghi-Sohi et al. [45] found that of the 4.3% missed diagnostic opportunities, 37% resulted in moderate to severe avoidable patient harm, often due to delays in follow-up investigations. Given the positive perception of workplace health screenings within the current study, future research should examine how these much-demanded approaches could be utilised to help alleviate participant concerns and support an already overburdened healthcare system. Working together to help support the health and wellbeing of the UK population should be the main consideration moving forward. Future endeavours should consider an approach where combining efforts and resources of organisations and health professionals is evident, helping combat barriers to evaluate, improve and understand the health of the population.

The qualitative data enhanced understanding of the perceived impact of a single workplace health screening session. Participants not only stated that they gained knowledge of their own health status but also understood how to monitor their health and implement changes to current behaviours, including modifying nutritional intake and physical activity participation and habits. Such changes were suggested by participants to have resulted in weight loss and reduced blood pressure, although this was not objectively recorded. It should be highlighted that these outcomes were recorded in a period of 2 to 10 months following the health screen (no repeat physiological measures were taken at this time). The present investigation focused on a single health screening session in a university staff population, with the provision focused on providing an overview of the individuals’ health status aligned to current guidelines, with individuals able to gain additional information if requested. The current remit was designed to evaluate the acceptability, feasibility and perceived value of single-session health screening approaches that could be utilised by employers outside of the university community, or by external organisations within a university setting. Given the cross-sectional nature of the current investigation, longitudinal qualitative and quantitative data is required to fully reflect the longstanding beneficial effects of workplace health screening services. Long-term behaviour-change strategies should also be considered to support health screening activities. Strudwick et al. [46], when considering mental health, suggested that workplace screening services are beneficial only if followed by structured access to care, illustrating the importance of integrated support systems post-screening. The recommendations offered by participants offer a basis for further screening development, particularly around the inclusion of longer-term follow-up screening opportunities, so that it is adapted to suit the needs of the workplace community [47]. The approach delivered within the current investigation, as supported by the qualitative data, suggests that these individual sessions displayed a perceived positive impact in understanding an individual’s health, with those individuals keen to remain involved in health screening services if future follow-up sessions were provided.

One of the key strengths of the present study is the mixed methodological approach in the evaluation of free workplace health screening for supporting employee wellbeing. The combination of qualitative and quantitative data allows greater understanding of the health of the individuals attending these sessions, and the perceived impact of the sessions for this population. It is important to note that of the 156 participants, the female consultation rates were vastly greater than males, accounting for 71% of the recruitment. Of the 67 individuals who completed the impact analysis, a similar percentage (75%, 69 individuals) of participants were female. This overrepresentation of females is in-line with the gender differences evident in healthcare and screening uptake [48]. That said, given that UK females consistently display greater comorbidity, with a higher burden of hypertension, elevated cholesterol, and self-reported mental health concerns [49,50], it is vital for females to continue to engage with these screening opportunities. Future research should consider the reasons for non-participation in health check-ups and reflect on approaches to increase health screening uptake in males to further understand the benefits of free workplace health screening on male workers within a university setting. Community involvement should also be considered given its recognition as a crucial component of effective healthcare delivery. Adopting practical, validated tools to evaluate and strengthen community involvement efforts should also be considered, given that such approaches have been shown to enhance trust, engagement, and overall health outcomes [47].

The current investigation was focused on evaluating the acceptability, feasibility, and perceived value of a single-session health screening approach within one institutional context. Consequently, several important limitations must be acknowledged. As with all single-institution observational studies, the findings are shaped by the culture, norms, and environmental characteristics of that setting, which may influence participant experiences and, in turn, affect study outcomes. This is particularly relevant given the established impact of workplace environments on health and wellbeing. If possible, future researchers should consider employing multi-site or cross-institutional study designs which would minimise the influence of any single organisational culture. Additionally, the voluntary nature of participation in both the health screening session and the subsequent qualitative data collection introduces the potential for volunteer bias. Individuals with heightened health awareness, whether due to existing concerns or an active interest in maintaining good health, may have been more likely to participate. Both motivational pathways were evident within the qualitative data. Given that qualitative feedback was provided in response to a free service, and positive evaluations may increase the likelihood of the service being offered again, responses may have been subject to social desirability bias. To strengthen future research, recruiting participants through stratified or random sampling processes, rather than relying solely on voluntary participation, would help improve representativeness. The current mixed-method cross-sectional investigation demonstrates some key limitations yet does provide evidence of the perceived benefits of such endeavours. The practical implications of the current study, as evidenced by the impact assessment data, support further efforts for integrated workplace health screening sessions to improve employee wellbeing and productivity, specifically at universities where campus services and staff have the expertise to conduct such assessments.

## 5. Conclusions

The current mixed-methods cross-sectional study with post-intervention survey provides an important understanding of the perceived benefits of free health screening in supporting employee wellbeing. Health screening sessions were identified to have a beneficial effect on supporting employees’ overall health awareness, with discussion of lifestyle changes in a population that displayed high levels of obesity, hypertension and prevalence of diabetes. The findings provide timely evidence that such an approach can be utilised in the current population as a well-received and feasible approach to support the understanding of health and wellbeing. Future research is encouraged and required through the application of these approaches to help reduce the burden of non-communicable diseases on healthcare systems via early preventative efforts. The current study highlights the potential of workplace health screening as a framework to foster a healthier, more productive work environment, ultimately contributing to long-term success.

## Figures and Tables

**Table 1:** Characteristics.

	Males	Females	Statistical Outcomes	Overall
**Demographic**				
Sample size	46 (29%)	110 (71%)		156 (100%)
Age (years)	42 ± 11 [95%CI: 39, 46]	43 ± 11 [95%CI: 41, 45]	*p* = 0.580 RBC = 0.06	43 ± 11 [95%CI: 41, 45]
Height (cm)	180 ± 7 [95%CI: 177, 182]	165 ± 6 [95%CI: 163, 166]	***p* < 0.001** **Cohen’s *d* = 2.24**	169 ± 9 [95%CI: 168, 171]
Weight (kg)	88 ± 19 [95%CI: 82, 94]	70 ± 15 [95%CI: 67, 73]	***p* < 0.001** **RBC = 0.56**	75 ± 18 [95%CI: 72, 78]
Current smoker	1 (2%)	2 (2%)	-	3 (2%)
**Family health status**				
Angina/Heart attack in first-degree relative under 60 years old	9 (20%)	25 (23%)	-	34 (22%)
Chronic kidney disease (stage 3, 4 or 5)	0 (0%)	3 (3%)	-	3 (2%)
Atrial fibrillation	0 (0%)	0 (0%)	-	0 (0%)
Blood pressure treatment	3 (7%)	4 (4%)	-	7 (4%)
Migraine	7 (15%)	27 (25%)	-	34 (22%)
Rheumatoid arthritis	0 (0%)	2 (2%)	-	2 (1%)
Systematic lupus erythematosus	0 (0%)	0 (0%)	-	0 (0%)
Atypical antipsychotic medication	5 (11%)	7 (6%)	-	12 (8%)
Severe mental illness	3 (7%)	6 (5%)	-	9 (6%)
Steroid use	1 (2%)	2 (2%)	-	3 (2%)
Erectile dysfunction	3 (7%)	-	-	3 (7%)
**Body composition**				
Waist-to-hip ratio (WHR)	0.78 ± 0.07 [95%CI: 0.77, 0.79]	0.88 ± 0.09 [95%CI: 0.86, 0.91]	***p* < 0.001** **RBC = 0.65**	0.81 ± 0.09 [95%CI: 0.79, 0.82]
Waist-to-height ratio (WHtR)	0.49 ± 0.07 [95%CI: 0.48, 0.51]	0.52 ± 0.07 [95%CI: 0.50, 0.54]	***p* = 0.02** **RBC = 0.24**	0.50 ± 0.07 [95%CI: 0.49, 0.51]
Body Mass Index (kg/m^2^)	27 ± 5 [95%CI: 26, 29]	26 ± 5 [95%CI: 25, 27]	*p* = 0.13 RBC = 0.15	26 ± 5 [95%CI: 25, 27]
**Cardiovascular health**				
Resting heart rate (bpm)	66 ± 11 [95%CI: 63, 69]	68 ± 10 [95%CI: 66, 70]	*p* = 0.330 Cohen’s *d* = 0.17	67 ± 11 [95%CI: 66, 69]
Systolic blood pressure (mmHg)	129 ± 16 [95%CI: 124, 134]	122 ± 15 [95%CI: 119, 125]	***p* = 0.010** **Cohen’s *d* = 0.44**	124 ± 16 [95%CI: 122, 126]
Diastolic blood pressure (mmHg)	83 ± 9 [95%CI: 80, 86]	81 ± 9 [95%CI: 79, 83]	*p* = 0.25 Cohen’s *d* = 0.20	82 ± 9 [95%CI: 80, 83]
Mean arterial blood pressure (mmHg)	98 ± 11 [95%CI: 95, 101]	95 ± 10 [95%CI: 93, 96]	*p* = 0.07 Cohen’s *d* = 0.32	96 ± 10 [95%CI: 94, 97]
**QRISK^®^3**				
Ten-year score	4.1 ± 6.2 [95%CI: 2.3, 6.0]	1.8 ± 1.9 [95%CI: 1.4, 2.2]	*p* = 0.64 Cohen’s *d* = 0.08	2.5 ± 3.9 [95%CI: 1.8, 3.5]
Relative risk	1.6 ± 1.4 [95%CI: 1.2, 2.1]	1.0 ± 0.5 [95%CI: 0.9, 1.1]	***p* < 0.001** **RBC = 0.41**	1.2 ± 0.9 [95%CI: 1.1, 1.3]

Note: BMI: Body mass index. RBC: Rank biserial correlation. Data is displayed as total (percentage) or mean ± 1 standard deviation [95% confidence intervals (95%CI)]. Bold highlights statistical significance (*p* ≤ 0.05).

**Table 2:** Characteristics questionnaire outcomes.

	Males	Females	Statistical Outcomes	Overall
**IPAQ-SF (met/week, [95%CI], classification)**	2120 ± 1480 [95%CI: 1693, 2548] Moderate activity	1978 ± 1413 [95%CI: 1713, 2243] Moderate activity	*p* = 0.499 RBC = 0.07	2020 ± 1429 [95%CI: 1795, 2245] Moderate activity
**OSPAQ (minutes, [95%CI], %)**				
Sitting	1611 ± 629 [95%CI: 1419, 1792] 69 ± 20%	1577 ± 558 [95%CI: 1473, 1681] 73 ± 19%	*p* = 0.655 RBC = 0.05	1587 ± 578 [95%CI: 1496, 1678] 72 ± 19%
Standing	317 ± 240 [95%CI: 248, 386] 14 ± 10%	238 ± 217 [95%CI: 197, 279] 12 ± 11%	***p* = 0.034** **RBC = 0.22**	261 ± 229 [95%CI: 226,297] 13 ± 11%
Walking	297 ± 250 [95%CI: 225, 369] 14 ± 11%	264 ± 232 [95%CI: 220, 307] 12 ± 9%	*p* = 0.433 RBC = 0.08	274 ± 157 [95%CI: 236, 311] 13 ± 10%
Heavy labour	63 ± 120 [95%CI: 28, 97] 3 ± 5%	56 ± 170 [95%CI: 24, 88] 3 ± 7%	*p* = 0.572 RBC = 0.05	58 ± 157 [95%CI: 33, 83] 2 ± 4%
**State Trait Anxiety Inventory**	27 ± 9 [95%CI: 25, 29] No or low anxiety	27 ± 10 [95%CI: 26, 29] No or low anxiety	*p* = 0.731 RBC = 0.04	27 ± 9 [95%CI: 26, 29] No or low anxiety
**PSQI (score, [95%CI], classification)**	7 ± 2 [95%CI: 7, 8] Poor sleep quality	8 ± 3 [95%CI: 8, 9] Poor sleep quality	*p* = 0.100 RBC = 0.17	8 ± 2 [95%CI:] Poor sleep quality
**Brunel Mood Scale Questionnaire**				
Anger	4 ± 4 [95%CI: 2, 5]	3 ± 4 [95%CI: 2, 4]	*p* = 0.170 RBC = 0.11	3 ± 4 [95%CI: 2, 4]
Tension	5 ± 4 [95%CI: 4, 6]	5 ± 5 [95%CI: 4, 6]	*p* = 0.933 RBC = 0.08	5 ± 5 [95%CI: 4, 6]
Depression	3 ± 3 [95%CI: 2, 4]	3 ± 4 [95%CI: 3, 4]	*p* = 0.453 RBC = 0.06	3 ± 4 [95%CI: 3, 4]
Vigour	13 ± 4 [95%CI: 12, 14]	10 ± 5 [95%CI: 9, 11]	***p* < 0.001** **RBC = 0.37**	11 ± 5 [95%CI: 10, 12]
Fatigue	6 ± 5 [95%CI: 5, 8]	8 ± 6 [95%CI: 7, 9]	***p* = 0.053** **RBC = 0.19**	8 ± 6 [95%CI: 7, 8]
Confusion	2 ± 3 [95%CI: 1, 3]	3 ± 4 [95%CI: 2, 4]	*p* = 0.20 RBC = 0.10	3 ± 4 [95%CI: 2, 4]
Happy	13 ± 4 [95%CI: 12, 14]	13 ± 4 [95%CI: 12, 13]	*p* = 0.393 RBC = 0.09	13 ± 4 [95%CI: 12, 13]
Calmness	10 ± 3 [95%CI: 9, 11]	8 ± 3 [95%CI: 8, 9]	***p* = 0.024** **RBC = 0.22**	9 ± 3 [95%CI: 8, 9]

Note: IPAQ-SF: International Physical Activity Questionnaire—Short Form; OSPAQ: Occupational Sitting and Physical Activity Questionnaire; STAI: State Trait Anxiety Inventory; RBC: Rank biserial correlation; PSQI: Pittsburgh Sleep Quality Index (classifications based on global PSQI score with ≤5 indicating good sleep quality and >5 indicating poor sleep quality [26]); BRUMS: Brunel Mood Scale Questionnaire. Data is displayed as mean ± 1 standard deviation, with 95% confidence intervals (95%CI) unless otherwise specified. Bold highlights statistical significance (*p* ≤ 0.05).

## Data Availability

Data are available upon reasonable request. All data relevant to the study are included in the article or uploaded as Supplementary Information.

## Supplementary Materials

The following supporting information can be downloaded at: https://www.mdpi.com/article/10.3390/healthcare14070912/s1.

## Abbreviations

The following abbreviations are used in this manuscript:

BMI	Body mass index
BRUMS	The Brunel Mood Scale Questionnaire
CI	Confidential interval
DBP	Diastolic blood pressure
ECG	Electrocardiogram
GP	General practitioner
HDL-C	High-density lipid cholesterol
HR	Heart rate
ICC	Inter-class correlation
IPAQ-SF	International Physical Activity Questionnaire—Short Form
MAP	Mean atrial blood pressure
MET	Metabolic equivalent
NHS	National Health Service
NICE	National Institute for Health and Care Excellence
OSPAQ	Occupational Sitting and Physical Activity Questionnaire
PSQI	Pittsburgh Sleep Quality Index
RBC	Rank biserial correlation
SBP	Systolic blood pressure
STAI	State Trait Anxiety Inventory
TC	Total cholesterol
UK	United Kingdom
WHR	Waist-to-hip ratio
WHtR	Waist-to-height ratio
