# Supplementary material for: From Assessment to Impact: How Workplace Health Screening Informs Staff Wellbeing in a University Setting
Source: Healthcare (Basel). 2026 Apr 1;14(7):912. doi: 10.3390/healthcare14070912 (PMC13072953; doi:10.3390/healthcare14070912)
Supplement: Supplementary file 1 [file healthcare-14-00912-s001.zip › healthcare-4137325-supplementary.pdf]

Supplementary Table S1: Overview of the qualitative impact thematic analysis including code counts for subtheme and overarching themes.

Codes and code count (*N*)	Lower order themes	Higher order themes
Friendly staff (n=23)<br>Staff put clients at ease (n=6)<br>Staff knowledge and expertise (n=7)<br>Staff were very professional (n=9)<br>Excellent service from staff (n=5)<br>Good experience overall (n=6)	Staff's skills were of great benefit to the overall experience	Positive experience with Healthy Heart Checks
Helpful information provided by staff (n=11)<br>Getting feedback about the assessment was appreciated (n=6)<br>Assessments were clearly explained (n=7)	Content and information provided	
The service was efficient (n=12)<br>Service was well organized (n=5)	Organization and efficiency	
Workplace service is more accessible (n=2)<br>The checks were accessible overall (n=2)<br>Checks were convenient in the workplace (n=2)<br>Less intimating than going to the GP (n=1)<br>Having checks across all sites (n=1)<br>Ensuring dates of the Checks are accessible to all (n=1)	Accessibility of Healthy Heart Checks	
Checks are a great benefit for staff (n=6)<br>Workplace health needs to be a priority (n=6)<br>Making employees feel valued (n=3)<br>The checks reduce absenteeism/presenteeism (n=4)<br>The workplace should fund the checks (n=2)<br>Showcasing University-ran initiatives (n=1)	Benefits as a workplace initiative	
Gaining a better understanding of one's health (n=5)<br>Potentially lifesaving information (n=5)<br>Service helped detect health concerns (n=7)<br>Getting positive results was reassuring (n=5)<br>Knowledge of health helped ease client concerns (n=2)<br>The checks helped increase health awareness (n=16)<br>Supporting health management (n=8)<br>Having the opportunity to examine concerning results (n=1)<br>Helped support understanding of genetic health risk factors (n=3)<br>Service has supported clients to improve their health (n=3)<br>Clients have taken something away from the service (n=3)<br>A useful reminder of the importance of regularly checking on health (n=3)<br>Some people already have a good awareness of health (n=3)	The overall positive impact of the assessment	Impact of Healthy Heart Checks
Changes implemented after the checks have already improved wellness (n=5)<br>Implementing healthy habits (n=12)<br>Maintaining current healthy behaviours (n=18)	Personal impact of Healthy Heart Checks on employee health	

Getting more active (n=11)<br>Better eating habits (n=8)<br>Weight loss (n=7)<br>Re-focusing health goals (n=3)<br>Helping to motivate individuals (n=4)<br>Looking after oneself more (n=2)<br>Improvements to overall wellbeing (n=1)<br>Receiving a diagnosis because of the checks (n=1)<br>Helping to reduce blood pressure (n=4)<br>Monitoring own health (n=7)<br>Reducing anxiety around one's health (n=1)<br>Scheduling a GP visit after receiving the results (n=6)		
Service helps to support referral to GP (n=1)<br>Easier to get the Healthy Heart Check done than with the GP (n=2)<br>Service provided additional information than what can be accessed through standard healthcare pathways (n=4)<br>Service fills a gap in current healthcare system (n=11)<br>Service supports participants in preventing ill health (n=7)<br>Helping to support what is currently available across health services (n=6)<br>Information could be used to follow up with GP (n=2)<br>Checks should not replace GP visits (n=1)	Helping to support health service efforts	
Would recommend the service to others (n=6)<br>Service should be offered permanently (n=3)<br>Clients support continuation of the service (n=9)<br>Service is incredibly valuable (n=2)<br>The health checks should be commercialized (n=3)<br>Utilization of broader university services (n=6)<br>Checks need to be regulated (n=1)<br>Considerations for the frequency of the checks (n=3)	Continuation of Healthy Heart Checks service	The future of Healthy Heart Checks
Overall wellbeing and lifestyle services (n=4)<br>Occupational health support (n=3)<br>Work related stress support (2)<br>Mental health support (n=1)	Wellness support	
Women's specific health checks (n=2)<br>Hormone and menopause specific service (n=5)<br>Bone mineral density assessment (n=3)	Women's specific health checks	
Fitness testing (n=3)<br>Nutritional support (n=2)<br>A physiotherapy service (n=2)<br>Ageing-related health check (n=2)<br>Anemia testing (n=2)<br>Vision checks (n=1)<br>Hearing checks (n=3)<br>Dental health (n=1)<br>In-depth assessment of the whole body (n=2)<br>Clients would attend any other health check (n=5)	Opportunities for other specific health services	
A follow-up appointment would be beneficial (n=4)		

Follow-ups would help track changes and progress (n=6)<br>Clients would prefer to discuss results with staff (n=3)<br>Follow-up sessions may be required for unusual results (n=1)<br>Receiving incentives for achieving goals based on health data (n=1)	Opportunities for follow-ups
Appointments were not regular enough to support attendance (n=1)<br>The checks are more suited to people who are not aware of their health status (n=1)<br>Some assessments felt rushed (n=2)<br>Some of the information/data received was confusing (n=1)<br>False alarm on data received (n=3)<br>Technical issues with some of the equipment (n=1)<br>The assessment structure was not always consistent (n=1)<br>Ordering of tests may need to be reconsidered (n=3)	Identified issues and challenges
